# Tissue-based associations of mammographic breast density with breast stem cell markers

**DOI:** 10.1186/s13058-017-0889-3

**Published:** 2017-08-29

**Authors:** Lusine Yaghjyan, Ethan Stoll, Karthik Ghosh, Christopher G. Scott, Matthew R. Jensen, Kathleen R. Brandt, Daniel Visscher, Celine M. Vachon

**Affiliations:** 1 Department of Epidemiology, College of Public Health and Health Professions and College of Medicine, University of Florida, 2004 Mowry Rd, Gainesville, FL 32610 USA; 2 Department of Pathology, Immunology and Laboratory Medicine, College of Medicine, University of Florida, 1600 SW Archer Road, Gainesville, FL 32610 USA; 3 Division of General Internal Medicine, Mayo Clinic College of Medicine, 200 First St SW, Rochester, MN 55902 USA; 4 Division of Biomedical Statistics and Informatics, Mayo Clinic College of Medicine, 200 First St. SW, Rochester, MN 55905 USA; 5 Department of Radiology, Mayo Clinic College of Medicine, 200 First St. SW, Rochester, MN 55905 USA; 6 Department of Anatomic Pathology, Mayo Clinic College of Medicine, 200 First St. SW, Rochester, MN 55905 USA; 7 Department of Health Sciences Research, Division of Epidemiology, Mayo Clinic College of Medicine, 200 First St. SW, Rochester, MN 55905 USA

**Keywords:** Breast density, Stem cell markers, Breast cancer risk, Immunohistochemistry, Staining extent

## Abstract

**Background:**

Mammographic breast density is a well-established, strong breast cancer risk factor but the biology underlying this association remains unclear. Breast density may reflect underlying alterations in the size and activity of the breast stem cell pool. We examined, for the first time, associations of CD44, CD24, and aldehyde dehydrogenase family 1 member A1 (ALDH1A1) breast stem cell markers with breast density.

**Methods:**

We included in this study 64 asymptomatic healthy women who previously volunteered for a *unique* biopsy study of normal breast tissue at the Mayo Clinic (2006-2008). Mammographically identified dense and non-dense areas were confirmed/localized by ultrasound and biopsied. Immunohistochemical analysis of the markers was performed according to a standard protocol and the staining was assessed by a single blinded pathologist. In core biopsy samples retrieved from areas of high vs. low density within the same woman, we compared staining extent and an expression score (the product of staining intensity and extent), using the signed rank test. All tests of statistical significance were two-sided.

**Results:**

A total of 64, 28, and 10 women were available for CD44, CD24, and ALDH1A1 staining, respectively. For all three markers, we found higher levels of staining extent in dense as compared to non-dense tissue, though for CD24 and ALDH1A1 the difference did not reach statistical significance (CD44, 6.3% vs. 2.0%, *p* < 0.001; CD24, 8.0% vs. 5.6%, *p* = 0.10; and ALDH1A1, 0.5% vs. 0.3%, *p* = 0.12). The expression score for CD44 was significantly greater in dense as compared to non-dense tissue (9.8 vs.3.0, *p* < 0.001).

**Conclusions:**

Our findings suggest an increased presence and/or activity of stem cells in dense as compared to non-dense breast tissue.

**Electronic supplementary material:**

The online version of this article (doi:10.1186/s13058-017-0889-3) contains supplementary material, which is available to authorized users.

## Background

Mammographic breast density is a well-established, strong predictor of breast cancer [1]. Mammographic breast density is a reflection of the amount of adipose, connective, and epithelial tissue in the breast. Although several studies investigated potential risk factors for breast density [2–6], the mechanisms by which breast density alters the breast cancer risk remain poorly understood. Breast tissue undergoes significant structural changes throughout a woman’s life [7]. The tissue architecture is maintained by a population of stem cells that have self-renewal capacity, which are essential for tissue repair and remodeling [8]. Tissue-specific stem cells serve as the source of the mature, functional cell types of a given tissue type, and have the capacity for self-renewal and the ability to regenerate its “home” tissue in its entirety [7, 9–12].

The mammary stem cells are a rapidly cycling cell population in the normal adult [12]. Larger mammary gland mass is expected to have a larger pool of mammary cells and be correlated with the number of mammary stem cells [13]. A stem cell hypothesis of breast carcinogenesis suggests that breast cancer development might be directly related to the size of the stem cell pool and its mitotic activity [14]. Perinatal variability in breast tissue (ranging from glands consisting of simple ductal systems to those with well-developed branching ducts complete with terminal lobules) suggests differences in the number and/or activity of stem cells between individuals, which may be important for adult breast cancer risk [9], potentially operating through the degree of mammographic breast density. According to the stem cell hypothesis of breast carcinogenesis, babies with greater birthweight would have a larger pool of stem cells that would determine their greater breast cancer risk. This hypothesis is supported by reports of greater breast cancer risk in women with larger birthweight in different populations [14, 15], higher concentration of umbilical cord blood cells expressing stem cell markers in babies with greater birthweight [9], and positive correlation between birthweight and mammographic breast density [9, 16]. Another established breast cancer risk factor, parity, has been consistently associated with decreased breast density [2, 6, 17, 18] and decreased breast cancer risk [19–22]. Emerging data also suggest decrease in the mammary stem cell numbers with increasing parity [22, 23] which would support an association of stem cells with breast density. Finally, in the mammary gland, stem cells are the only cell subpopulation that has capacity to accumulate all the oncogenic alterations [7].

There are limited data on expression of stem cell markers in healthy breast tissue, in particular the epithelium and stroma of normal breast tissue in asymptomatic women. Well-characterized stem cell markers CD44 and CD24 have been linked to younger age at diagnosis, higher odds of unfavorable tumor characteristics, including triple-negative receptor status (estrogen, progesterone, and human epidermal growth factor receptor 2 (HER2)), and distant metastasis [24–26]. Another stem cell marker, aldehyde dehydrogenase family 1 member A1 (ALDH1A1), is correlated with poor prognosis and chemotherapy-resistant breast cancer [26–29]. Whether mammographic breast density reflects the number and expression of stem cells in the breast is unknown. To fill this knowledge gap, we examined associations between the expression of CD44, CD24, and ALDH1A1 in normal breast tissue samples retrieved from the areas of high and low breast density in the same woman.

## Methods

### Study design and population

This study included 64 asymptomatic healthy women who previously volunteered for a study of normal breast tissue at the Mayo Clinic (Rochester, MN, USA) between 2006 and 2008. Details of this study have been described previously [30, 31]. Briefly, eligible women were aged 40 years or older with no personal history of breast cancer, a normal screening mammogram within 6 months of biopsy, and mammographically dense and non-dense areas identified on the mammogram, and confirmed and localized by ultrasound, that could be biopsied for the study. Women were ineligible if they were currently using postmenopausal hormones, oral contraceptives, other endocrine therapy, or anticoagulants. Other exclusions were history of bleeding complications or allergy to local anesthetic agents. All participants completed a self-administered questionnaire on breast cancer risk factors including age at menarche, age at first parity, family history of breast cancer in a first-degree relative, use of oral contraceptives or postmenopausal hormone therapy, number of breast biopsies, and prior history of atypical hyperplasia. There were in total 64 women available for this investigation; the majority of these women were included in our prior investigations and the same protocols were used for acquisition and sectioning of tissue [30, 31]. This study was approved by the Mayo Clinic, Rochester and the University of Florida Institutional Review Boards. Written consent was obtained from all participants.

### Ascertainment of dense and non-dense cores

In the original core biopsy study, the study radiologist identified areas of high and low density in the right breast (the upper outer quadrant in most cases) using mammogram films taken within 6 months [30, 31]. If the patient had a previous benign surgical biopsy in the right breast, then the left breast was selected for biopsy. The areas of mammographically dense and non-dense tissue in the upper-outer region of the breast (≥50% and < 25% density represented by fibroglandular elements, respectively), were identified by an experienced breast radiologist, then localized by ultrasound in a similar fashion to routine clinical practice where mammographic findings are further evaluated with ultrasound [30, 31]. Sonographically, the dense tissue selected for biopsy was either homogenously hyperechoic (relative to subcutaneous breast fat) or a heterogenous mixture of hyperechoic tissue and hypoechoic ducts. Sonographically, the non-dense tissue selected for biopsy was isoechoic to subcutaneous breast fat. Using a 14-gauge needle, an ultrasound-guided core-needle biopsy was performed in the identified dense and non-dense regions. Four cores were taken from each region; three cores were formalin fixed and embedded in one paraffin block and the remaining core was frozen. Thirty serial sections were cut from each paraffin-embedded block. Slides (sections 1, 15, and 30) cut from both the dense and non-dense paraffin-embedded blocks were stained with hematoxylin and eosin (H&E) and histologically examined to ensure that sections contained benign tissue [31]. In our previous study using these samples [30], all H&E slides from dense and non-dense areas were assessed by an experienced pathologist for the type of benign breast tissue, which was classified as non-proliferative disease, proliferative disease without atypia, and atypical hyperplasia using standard criteria described by Dupont et al. [32, 33]. The prevalence of proliferative changes among 59 women in our prior study was higher in slides from dense areas as compared to non-dense areas [30]. Of the slides from dense areas, 11.9% contained proliferative disease without atypia vs. 0% in the non-dense areas. The rest of the slides from both dense (88.1%) and non-dense areas (100%) were represented by non-proliferative tissue. Presence of atypical hyperplasia was assessed on all slides from 64 women. None of the slides from dense or non-dense areas showed presence of atypia.

Due to the design of our study, only patients with samples taken both from regions of dense and non-dense tissue in the same breast were considered, and these were stained and analyzed. Thus, the histologic assessment and staining were not performed on sections from patients who only had dense or non-dense tissue. Eligible women and those who were excluded from this study had similar breast imaging reporting and data system (BI-RADS) breast density.

### Immunohistochemical analysis and interpretation

Immunohistochemical analysis of the markers on 5-μm tissue sections was performed at the University of Florida Pathology Core Laboratory according to a standard protocol and using commercially available antibodies (DAKO AutostainerPlus, CD44 (DAK) 1:25 dilution; CD24 (Abcam) 1:200 dilution; and ALDH1A1 (Abcam) 1:300 dilution). For this investigation, we had tissue sections available for 64, 28, and 10 women to stain for CD44, CD24, and ALDH1A1, respectively. Staining for each of the tissue markers was performed on a separate section from the dense and non-dense areas of the woman’s breast (sections 5, 12, and 13 from the original core; total of 128 slides for CD44, 56 for CD24, and 20 for ALDH1A1). Slides were de-paraffinized with xylene and rehydrated through decreasing concentrations of ethanol to water, including an intermediary step to quench endogenous peroxidase activity (3% hydrogen peroxide in methanol). Slides were transferred to 1 × Tris-buffered saline-Tween (TBS-T).). For heat-induced antigen retrieval, sections were heated in a steamer while being submerged in Citra (Biogenex, Fremont, CA, USA) or Trilogy (Cell Marque, Rocklin, CA, USA) for 30 minutes. Slides were subsequently rinsed in 1 × TBS-T and incubated with a universal protein blocker Sniper (Biocare Medical, Walnut Creek, CA, USA) for 15 minutes and then rinsed in 1 × TBS-T and co-incubated in primary antibody ALDH1A1 or CD24 or CD44 for 1 hour. Next, slides were rinsed in 1 × TBS-T followed by application of conjugated secondary antibody: Mach 2 goat anti-rabbit horse (or mouse) radish peroxidase-conjugated (Biocare Medical, Walnut Creek, CA, USA) for 30 minutes. Antibodies were detected by incubating slides in 3’3’ diaminobenzidine (Vector Laboratories Inc., Burlingame, CA, USA) for 4 minutes. Slides were counterstained with hematoxylin (Biocare Medical, Walnut Creek, CA, USA) 1:10 for 3 minutes and mounted with Cytoseal XYL (Richard-Allen Scientific, Kalamazoo, MI, USA).

**Fig. 1:**
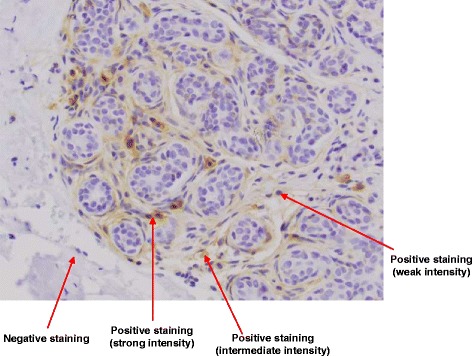
Example of positive and negative staining for CD44

The percentage of total immunoreactivity and staining intensity for each marker was assessed by a single pathologist using the Olympus BX43. Any cellular staining pattern (e.g. nuclear, cytoplasmic or membranous) was considered positive. Staining extent was quantified as percentage of the area occupied by positively stained cells out of the total tissue area in the slide. Average intensity of the staining in each slide was categorized as weak (1+), intermediate (2+) or strong (3+) (Fig. 1). The composition of the breast tissue was evaluated as the proportion of epithelial, stromal, and adipose tissue elements. Additional staining images are provided in Additional file 1.

### Statistical analysis

Data on stem cell marker expression are presented as means and standard deviations. First, the analysis for a given marker was restricted to the subjects with ≥ 1% presence of either epithelium or stroma in both dense and non-dense cores stained for this marker. In a secondary analysis, we further restricted the comparison for a given marker to the subjects with ≥ 10% of combined epithelial and stromal elements in both dense and non-dense sections stained for this marker. The correlation between proportion of epithelial and stromal area across the sections stained for three markers was very high (>0.9). The signed rank test was used to compare the expression of each stem cell marker in core biopsy samples that were retrieved from areas of high vs. low density. We separately examined staining extent and an expression score that represented the product of staining intensity and extent.

In a secondary analysis, we compared expression of the markers in dense and non-dense areas using repeated measures generalized linear models while adjusting for proportion of epithelial tissue, proportion of stroma, and proportion of total epithelial and stromal area. In this analysis, the expression of the markers was first square-root-transformed to improve normality and then converted into one standard deviation scale for ease of comparison across markers with differing scales. These models used a freely estimable covariance structure to account for correlation between dense and non-dense areas within each woman. Analyses were performed using SAS software (version 9.4; SAS Institute, Cary, NC, USA). All tests of statistical significance were two-sided (with H_0_ = no difference between dense and non-dense tissue) and the level of statistical significance was set at *p* < 0.05.

## Results

**Fig. 2:**
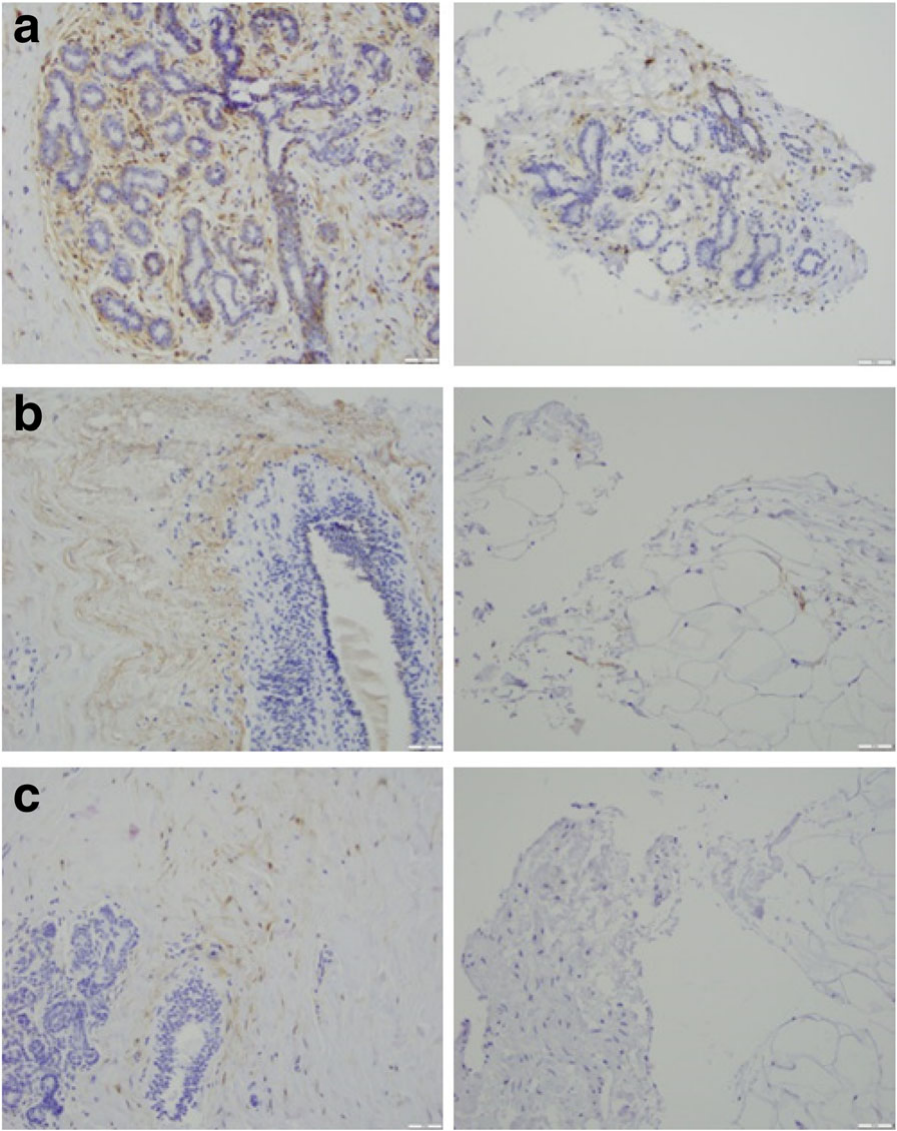
Immunohistochemical staining for CD44 (**a**), CD24 (**b**), and aldehyde dehydrogenase family 1 member A1 (ALDH1A1) (**c**) in sections from dense (left) and non-dense (right) areas within the same woman

**Fig. 3:**
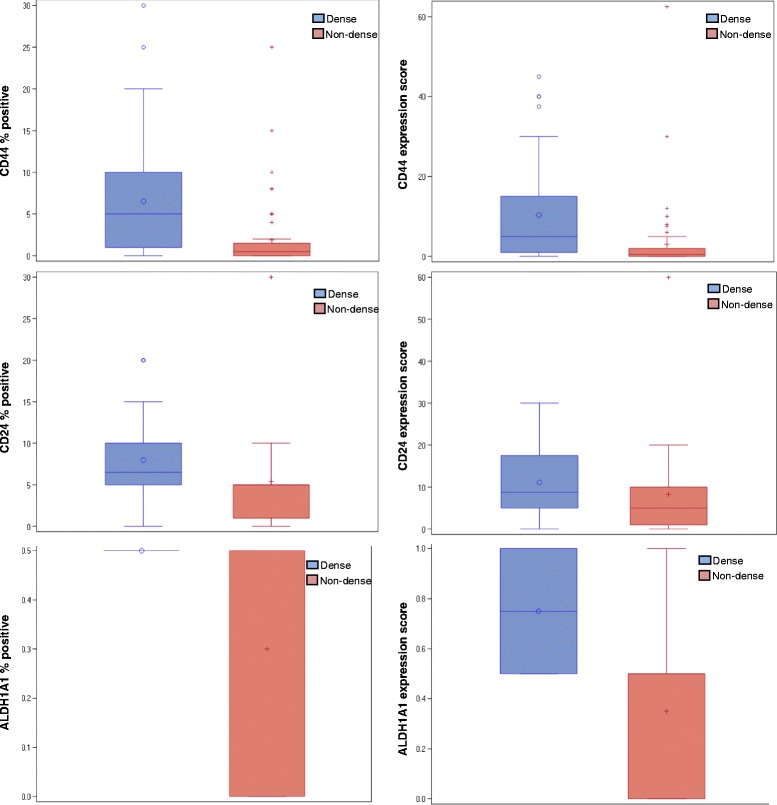
Distribution of staining extent and expression score for CD44, CD24, and aldehyde dehydrogenase family 1 member A1 (ALDH1A1) in tissue samples from mammographically dense and non-dense areas

The characteristics of the 64 women in this study are presented in Table 1. All women were Caucasian and the sample included both premenopausal (n = 22; 34%) and postmenopausal (n = 42; 66%) women, with mean age 51 years (range 40–79). The proportions of different tissue elements (epithelial, stromal, and adipose) in the paired samples from dense and non-dense areas are presented in Additional file 2 Table S1. Figure 2 represents pairs of samples from dense and non-dense areas of the breasts of the same woman stained for each of the markers. For all three markers, we found higher levels of staining extent in dense as compared to non-dense tissue, though for CD24 and ALDH1A1 the difference was not statistically significant (6.3% vs. 2.0% for CD44, *p* < 0.001; 8.0% vs. 5.6% for CD24, *p* = 0.10; and 0.5% vs. 0.3% for ALDH1A1, *p* = 0.12) (Table 2 and Fig. 3). The expression score (product of extent and intensity) for CD44 was significantly greater in dense as compared to non-dense tissue (9.8 vs. 3.0, *p* < 0.001) (Table 2). The average expression scores for CD24 and ALDH1A1 appeared to be greater in dense as compared to non-dense tissue, but these differences were not statistically significant (11.1 vs. 8.5, *p* = 0.40 and 0.8 vs. 0.4, *p* = 0.06, respectively) (Table 2). These differences in staining extent and expression score for all three markers were similar when the analysis was restricted to subjects with ≥ 10% of epithelial and stromal elements in both dense and non-dense cores (Table 3).

**Table 1:** Characteristics of 64 women in the study

Characteristic	Mean (STD) or number (percentage)
Age at mammogram (continuous, years)	51.1 (9.6)
Body mass index (continuous, kg/m^2^)	27.0 (4.7)
Parity and age at first child’s birth	
Nulliparous	10 (16%)
1–4 children with age at first birth ≤ 24 years	24 (38%)
1–4 children with age at first birth of 25–29 years	13 (20%)
1–4 children with age at first birth of ≥ 30 years	15 (23%)
≥ 5 children with age at first birth of < 25 years	0 (0%)
≥ 5 children with age at first birth of ≥ 25 years	2 (3%)
Menopausal status	
Premenopausal	22 (34%)
Postmenopausal	42 (66%)
Postmenopausal hormone use	
Never	28 (67%)
Past	13 (31%)
Current	0 (0%)
Unknown	1 (2%)
Family history of breast or ovarian cancer in first-degree relatives^a^	26 (43%)

*STD* standard deviation

^a^Data were missing for 4 women

**Table 2:** Subset of subjects with ≥ 1% epithelium or ≥ 1% stroma in both dense and non-dense cores

Marker expression measure	Dense		Non-dense		*P* for difference*
	Number	Mean (STD)	Number	Mean (STD)	
CD44					
Staining extent	59	6.3 (6.7)	59	2.0 (4.2)	<0.001
Expression score (extent x intensity)	59	9.8 (11.3)	59	3.3 (9.1)	<0.001
CD24					
Staining extent	27	8.0 (6.0)	27	5.6 (5.8)	0.10
Expression score (extent x intensity)	27	11.1 (8.6)	27	8.5 (11.5)	0.40
ALDH1A1					
Staining extent	10	0.5 (0)	10	0.3 (0.3)	0.12
Expression score (extent x intensity)	10	0.8 (0.3)	10	0.4 (0.3)	0.06

*STD* standard deviation, *ALDH1A1* aldehyde dehydrogenase family 1 member A1

*P value from signed rank test for alternative hypothesis of associations vs. null hypothesis of no association with significance level of 0.05

**Table 3:** Subset of subjects with sum of epithelium and stroma ≥ 10% in both dense and non-dense cores

Marker Expression measure	Dense		Non-dense		*P* for difference*
	Number	Mean (STD)	Number	Mean (STD)	
CD44					
Staining extent	34	7.4 (7.6)	34	3.3 (5.1)	0.009
Expression score (extent x intensity)	34	11.8 (13.0)	34	5.4 (11.6)	0.01
CD24					
Staining extent	18	8.7 (6.5)	18	6.5 (6.8)	0.36
Expression score (extent x intensity)	18	11.5 (9.3)	18	10.2 (13.7)	0.45
ALDH1A1					
Staining extent	6	0.5 (0)	6	0.3 (0.3)	0.17
Expression score (extent x intensity)	6	0.8 (0.3)	6	0.3 (0.3)	0.09

*STD* standard deviation, *ALDH1A1* aldehyde dehydrogenase family 1 member A1

*P value from signed rank test for alternative hypothesis of associations vs. null hypothesis of no association with significance level of 0.05

In a univariate analysis, the staining extent and expression score for CD44 and ALDH1A1 were significantly associated with greater breast density (Table 4). In the secondary analysis with adjustment for epithelial area, the difference in the expression patterns was no longer significant, except for ALDH1A1 expression. The association patterns were no longer evident with additional adjustment for either proportion of stroma or combined proportion of epithelial and stromal elements. As stem cell markers predominantly express in epithelial and stromal elements in the breast [34], this change in association pattern is expected with additional adjustment for epithelial and stromal area (Table 3).

**Table 4:** Association of stem cell markers with breast density, adjusted for epithelial/stromal tissue, regression coefficient (standard error) for dense vs. non-dense area

Marker Expression measure^a^	Unadjusted	Adjusted for percentage epithelium	Adjusted for percentage epithelium and stroma	Adjusted for percentage stroma
CD44				
Staining extent	0.95 (0.14)*	0.01 (0.09)	−0.29 (0.14)	0.30 (0.26)
Expression score (extent x intensity)	0.89 (0.14)*	−0.04 (0.10)	−0.25 (0.16)	0.35 (0.26)
CD24				
Staining extent	0.44 (0.29)	0.12 (0.30)	−1.36 (0.43)*	−0.65 (0.48)
Expression score (extent x intensity)	0.37 (0.29)	0.09 (0.30)	−1.45 (0.46)*	−0.75 (0.49)
ALDH1A1				
Staining extent	0.97 (0.40)*	0.65 (0.40)	−0.31 (0.71)	0.18 (0.76)
Expression score (extent x intensity)	1.11 (0.41)*	0.95 (0.40)^b^	0.48 (0.63)	0.61 (0.74)

*ALDH1A1* aldehyde dehydrogenase family 1 member A1

^a^All markers transformed using square root and put on one standard deviation scale

^*^Significant at 0.05

## Discussion

Our study provides the first evidence that the expression of stem cell markers is increased in the areas of dense breast tissue as compared to low density area within the same woman. These findings suggest that stem cell markers could partially explain the underlying molecular mechanisms behind the associations of high breast density with increased breast cancer risk.

Breast tissue is very dynamic and undergoes significant remodeling and structural changes during puberty, pregnancy, lactation, and involution [7]. Breast stem cells are essential for the continuous tissue remodeling and maintenance of the breast tissue architecture. The potential association between stem cells and breast density is supported by findings from previous studies that demonstrate consistent associations between epidemiologic risk factors that influence breast stem cell activity and breast density. For example, birthweight is positively associated with higher breast density and breast cancer risk in previous studies [14–16, 35–37] and these associations are explained by higher insulin-like growth factor 1 (IGF-1) levels during adulthood in women with greater birthweight [38]. The IGF-1 pathway is implicated in epithelial-to-mesenchymal transition (EMT), a naturally occurring process for tissue remodeling in both normal and cancerous tissue. Cells undergoing EMT also acquire stem-cell-like characteristics suggesting an overlap between EMT and stem cell mechanisms [39]. In contrast, greater childhood and adolescent body fat is associated with an increase in sex steroid hormones in heavier girls, early breast tissue differentiation and reduction in the stem cell pool, and lower levels of IGF-1 in adulthood [40–42]. Consistently, greater adolescent body size is associated with decreased breast density and reduced risk of breast cancer [41, 43–47]. Finally, emerging data suggests a decrease in the stem cell pool with increasing parity [22, 23, 48], which is also associated with lower breast density and reduced breast cancer risk [2, 6, 18, 19].

Our findings suggest greater expression of stem cell markers in areas of dense as compared to non-dense breast tissue. As expected, these differences were attenuated after additional adjustment for proportion of epithelial or stromal elements and were further attenuated after adjustment for the combined area of epithelial and stromal tissue. Previous literature suggests that stem cell markers are expressed in epithelium and stroma [34]. In our study, expression of all three markers was more apparent in tissue sections with a larger proportion of epithelium. We observed strong correlation between staining positivity and the proportion of epithelium (correlation coefficients 0.83, 0.31, and 0.50 for CD44, CD24, and ALDH1A1, respectively), and moderate correlation with the proportion of stroma for all three markers (0.55, 0.38, and 0.51, respectively). The staining for all three markers was detected even in some sections with no epithelium, but a large proportion of stromal tissue, suggesting that expression in the stromal tissue is also important. These findings could suggest that breast-density-associated increase in breast cancer could potentially result from greater presence of cells with stem-like properties in dense breast tissue. However, we cannot directly determine the specific location of the increased expression as being within the stroma or the epithelium.

We examined, for the first time, the association of CD44, CD24, and ALDH1A1 expression with dense and non-dense areas of the breast in healthy, asymptomatic women. We utilized a unique tissue resource from healthy volunteers who have provided core biopsies of dense and non-dense regions allowing the comparison of stem cell markers within a woman. This type of resource with healthy tissue sampled specifically from dense and non-dense regions is unique and allows for comparison of tissue markers within the same breast. Our study has a few limitations. Marker staining was assessed by a single expert pathologist. We did not count the number of different cell types in the sections but rather estimated the proportion of epithelial, stromal, and fat tissue area out of the total tissue area. These proportions were further used for adjustment for tissue composition in our secondary analysis. Although efforts were taken, the pathologist cannot be completely blinded to the dense or non-dense status of the sections due to the general composition of dense tissue being more likely to contain larger amounts of stromal and epithelial cells as compared to non-dense tissue. However, as the pathologist was not aware of the research hypothesis, it is unlikely that the results of the staining readings were influenced by the knowledge of the tissue composition. Validation studies have demonstrated very good inter-observer agreement in staining assessment by different experts and between automated and pathologist readings [49–51]. Consistently, in clinical practice, reading of the staining results for several markers is performed by only one pathologist and not by consensus of multiple pathologists. Further, use of a single reader eliminates inter-rater variability. In the core biopsy study, the areas of dense and non-dense tissue were identified by a single radiologist. Previous studies demonstrate very good agreement in breast density assessment by different radiologists [52–54]. Finally, in our study, initial assessment of density on a mammogram was further confirmed with ultrasound.

We had tissue sections from only 28 and 10 women for CD24, and ALDH1A1, respectively. However, because of the paired nature of the dense and non-dense sections, this sample size was sufficient to detect a 0.37 standard deviation difference in CD44, 0.5 standard deviation difference in CD24 and a 1 standard deviation difference in AHDH1A1 between dense and non-dense tissue, with 80% power, thus making our results informative. Some recent studies indicate that staining intensity can be affected by both storage time and variability in processing [55]. As our samples were collected over 3 years, the influence of storage time on staining intensity results could not be ruled out completely. However, due to the paired nature of the cores, it would likely result in attenuation of any associations between dense and non-dense tissue. In contrast to the expression score results that incorporate information on staining intensity, the comparison of staining extent has consistently demonstrated greater expression of all three markers in dense as compared to non-dense areas. Finally, in this preliminary investigation, we did not have sufficient power to examine associations between stem cell markers and breast density by the status of postmenopausal hormone use, family history of breast cancer, or other important risk factors known to be associated with density.

## Conclusions

This study provides the first evidence on the expression of stem cell markers in normal breast tissue from healthy women and contributes to the understanding of potential pathways by which breast density may influence breast cancer. As activity of breast stem cells is potentially modifiable by immunotherapy, modulation of specific target pathways and some therapeutic interventions [56–58], if an association between stem cells and density is confirmed in subsequent studies, these findings may pave a way for new therapeutic interventions to reduce breast density and breast cancer risk in high-risk women with dense breasts. Importantly, strategies aimed at breast density reduction will apply to a large segment of women (~50%) with dense breasts. Reduced breast density will not only decrease subsequent breast cancer risk but will also improve mammography performance (by improving sensitivity in less dense breasts) and facilitate detection of breast cancer at an early and curable stage.

## Additional files

Additional file 1: Figure S1. Examples of staining in sections with different tissue composition. (DOCX 12106 kb)

Additional file 2: Table S1. Distribution of the proportion of stroma, epithelium and adipocytes in the dense and non-dense matched core biopsies of the breasts of 64 women. (DOCX 19 kb)

## Abbreviations

ALDH1A1: Aldehyde dehydrogenase family 1 member A1
EMT: Epithelial-to-mesenchymal transition
HER2: Human epidermal growth factor receptor 2
IGF-1: Insulin-like growth factor 1
TBS-T: Tris-buffered saline-Tween
