# Supplementary material for: Tissue-based associations of mammographic breast density with breast stem cell markers
Source: Breast Cancer Res. 2017 Aug 29;19:100. doi: 10.1186/s13058-017-0889-3 (PMC5576318; doi:10.1186/s13058-017-0889-3)

**Supplemental figure 1**. Examples of staining in sections with different tissue composition.

A. Dense tissue with only 5% epithelium and 5% stroma


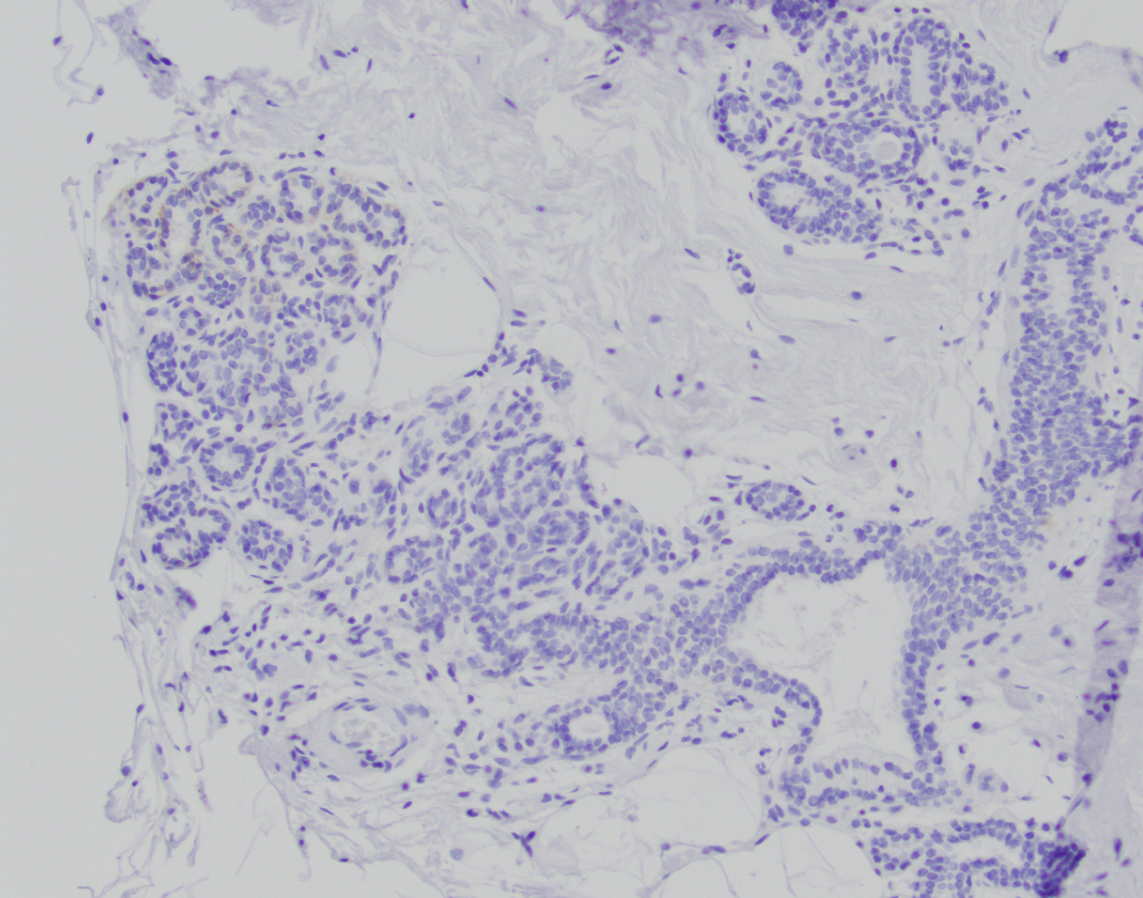


B. Dense tissue with 25% epithelium and 5% stroma


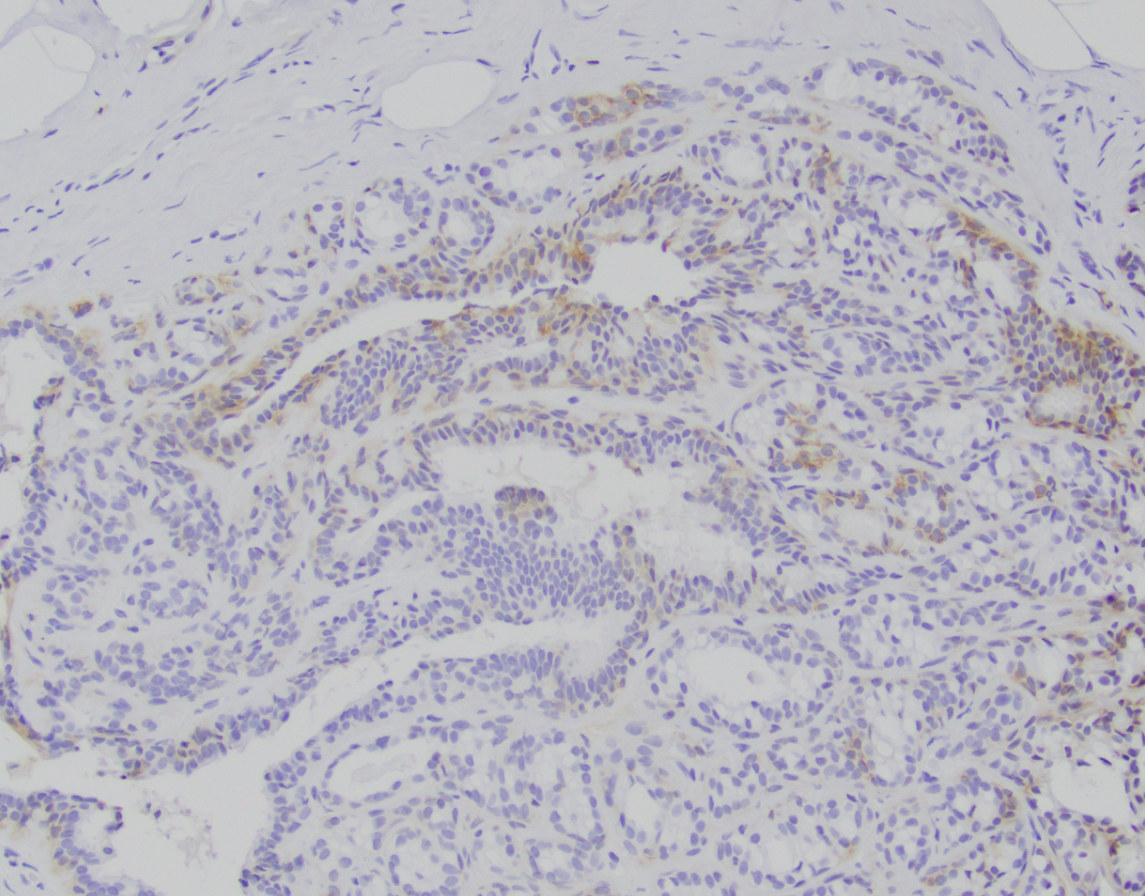


C. Non-dense tissue with 0% epithelium and stroma


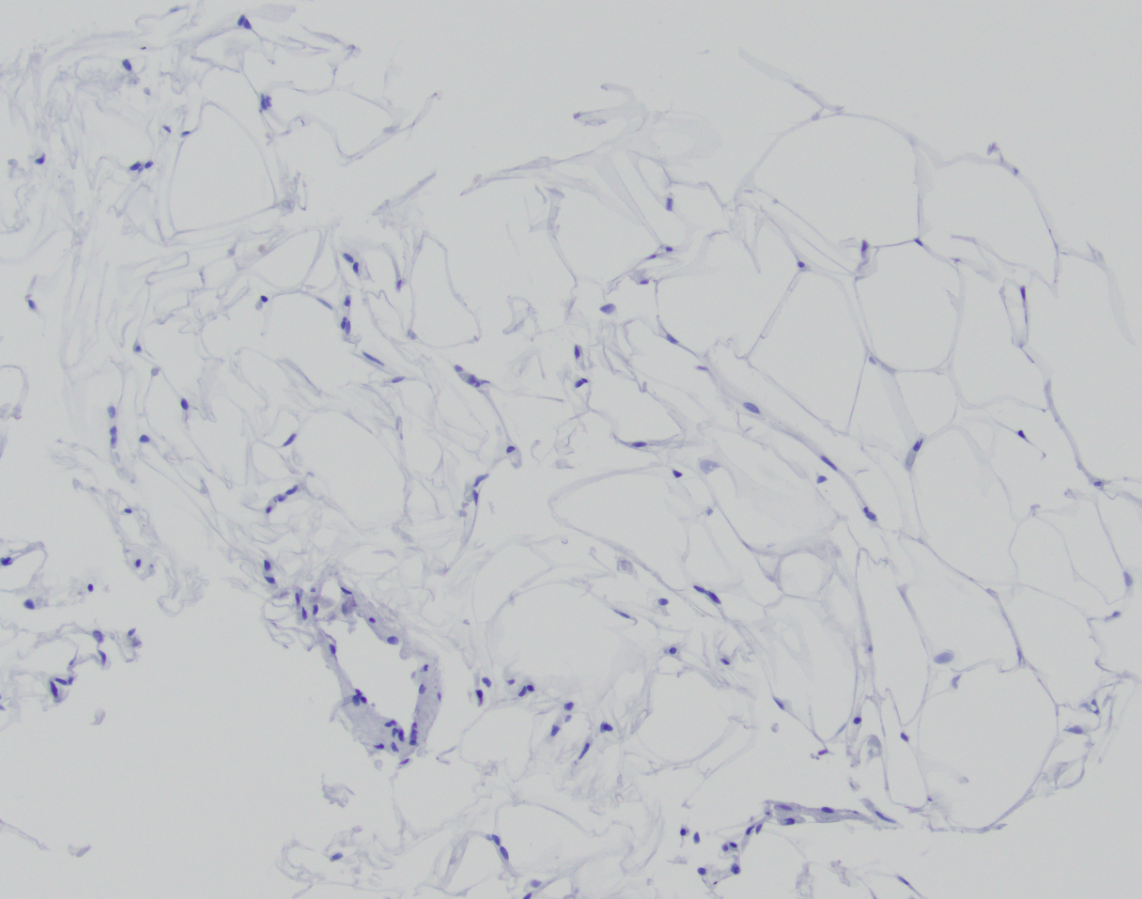


D. Non-dense tissue with 5% epithelium and 5% stroma


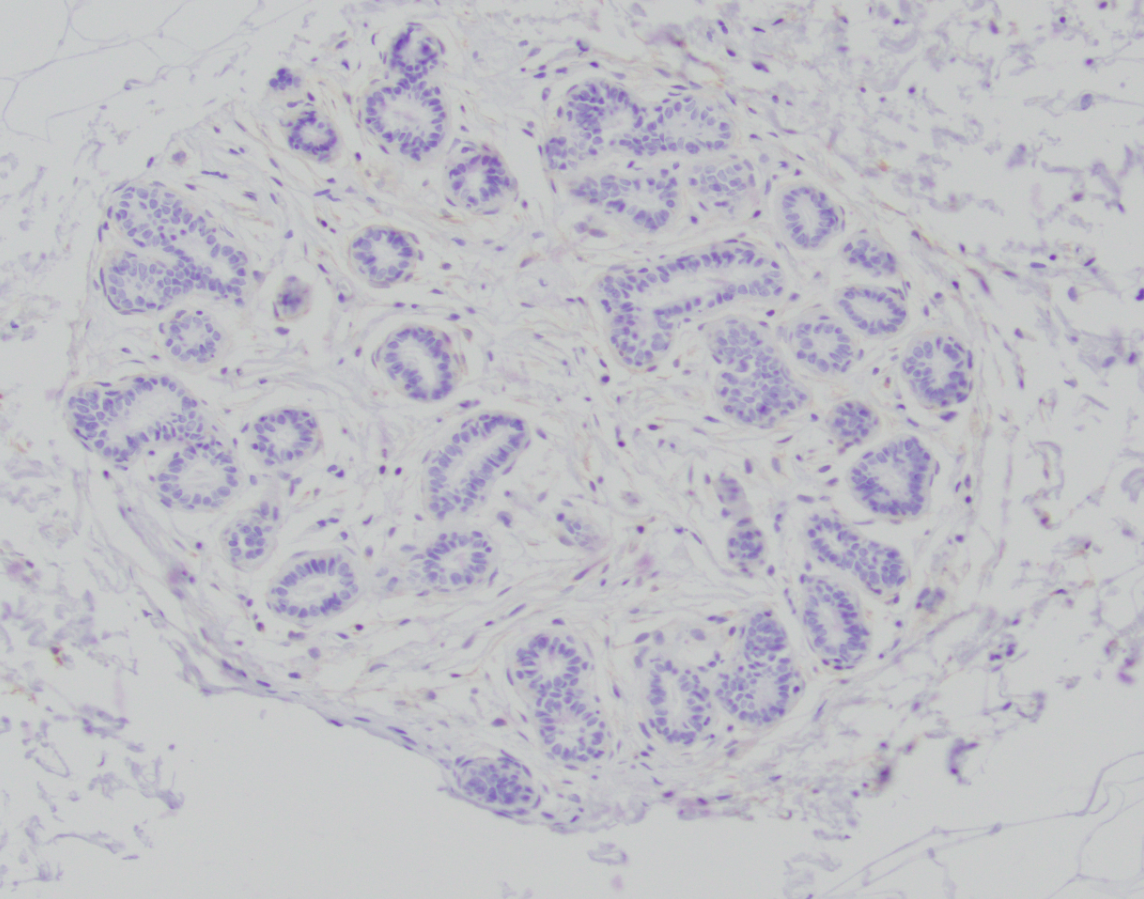


E. Dense tissue with 9% epithelium and 95% Stroma


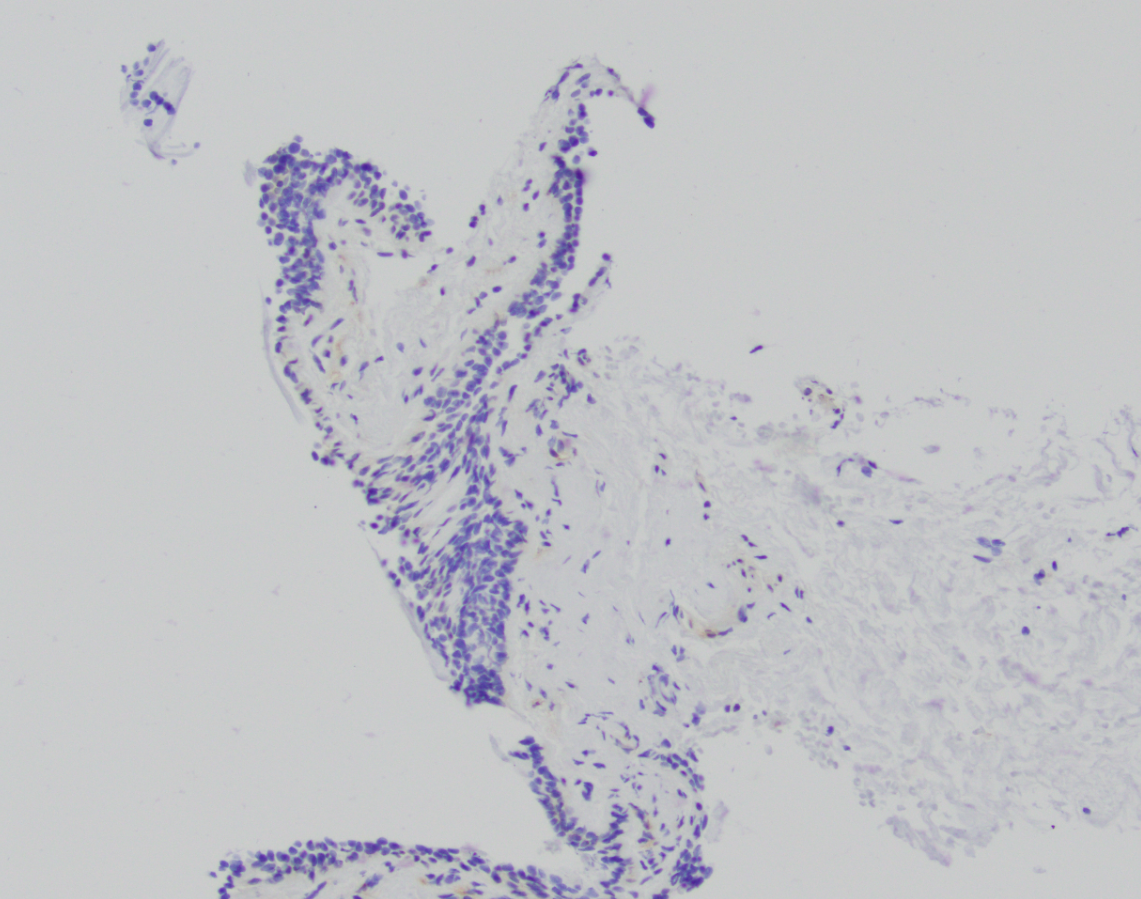


F. Dense tissue with 25% epithelium and 70% stroma


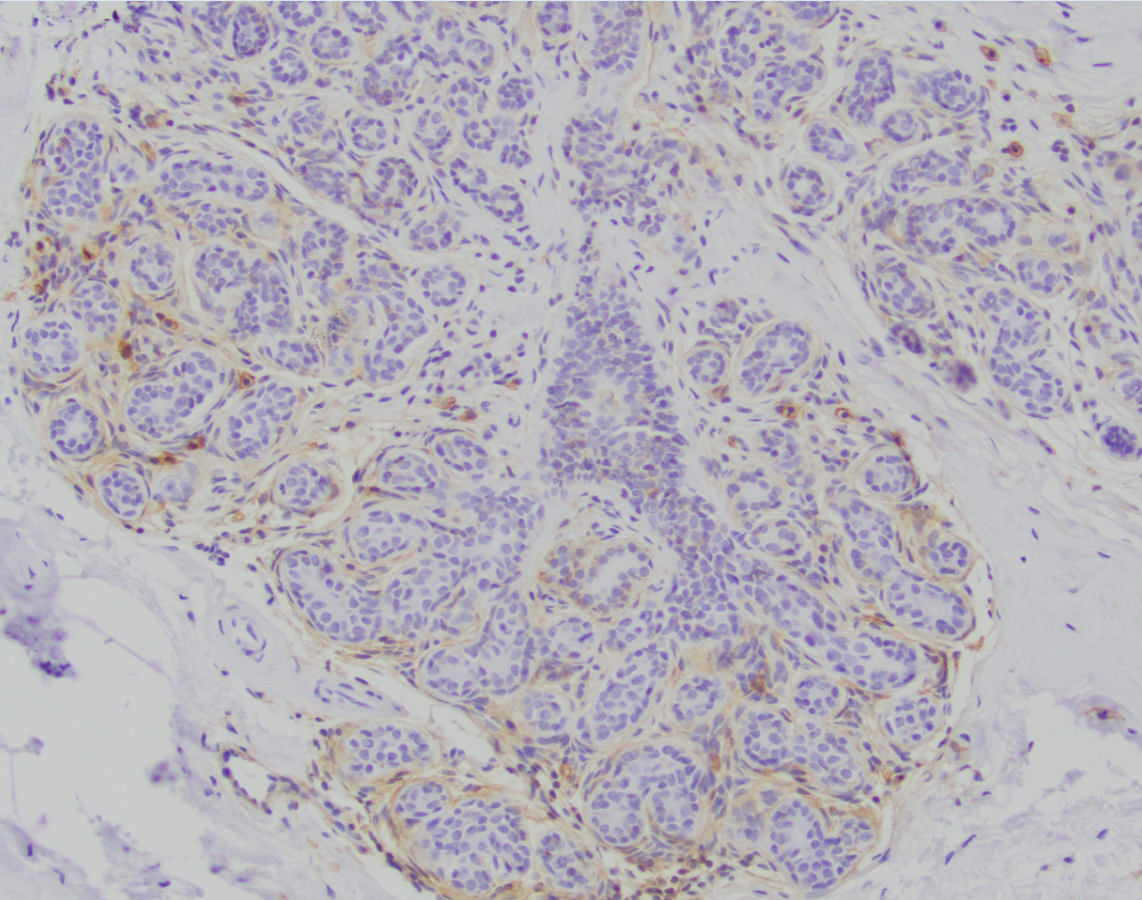


G. Non-dense tissue with 25% epithelium and 25% stroma


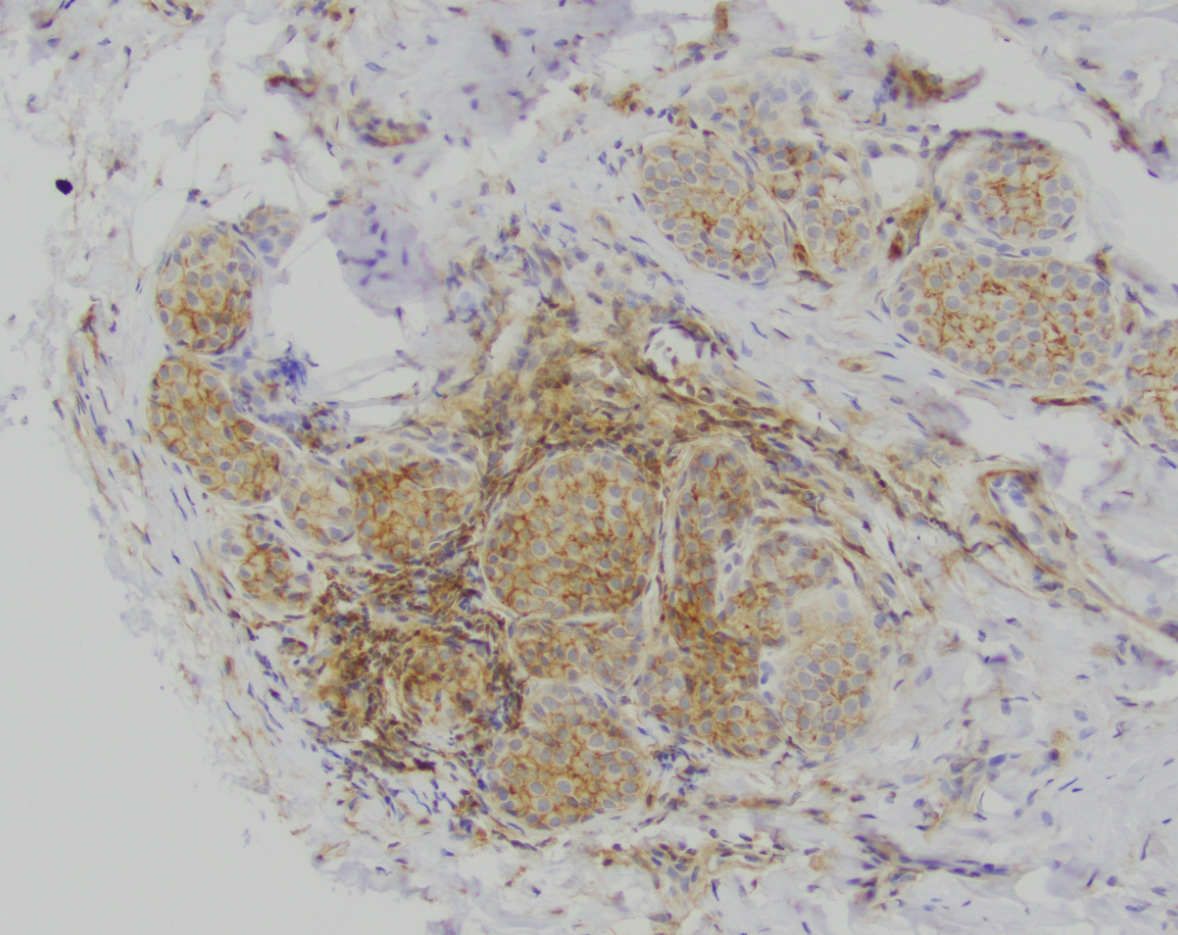


H. Non-dense tissue with 1% epithelium and 49% stroma


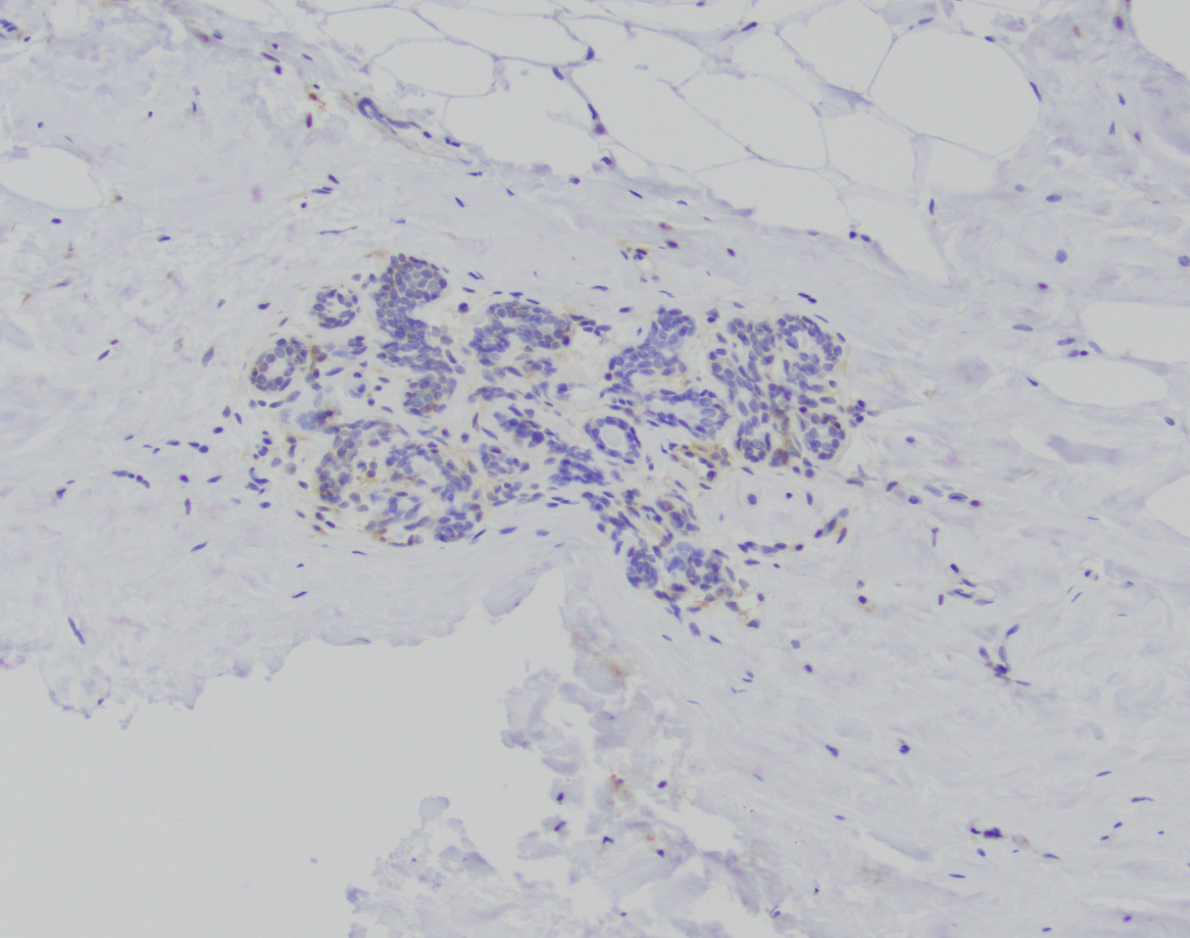

Supplement: Supplementary file 1 — Examples of staining in sections with different tissue composition. (DOCX 12106 kb) [file 13058_2017_889_MOESM1_ESM.docx]
