# Supplementary material for: Tissue-based associations of mammographic breast density with breast stem cell markers
Source: Breast Cancer Res. 2017 Aug 29;19:100. doi: 10.1186/s13058-017-0889-3 (PMC5576318; doi:10.1186/s13058-017-0889-3)
Supplement: Supplementary file 2 — Distribution of the proportion of stroma, epithelium and adipocytes in the dense and non-dense matched core biopsies of the breasts of 64 women. (DOCX 19 kb) [file 13058_2017_889_MOESM2_ESM.docx]

| **Supplementary table S1.** Distribution of the proportion of stroma, epithelium and adipocytes in the dense and non-dense matched core biopsies of the breasts of 64 women | | | | | | | |
| --- | --- | --- | --- | --- | --- | --- | --- |
| Cell type | Proportion of | Proportion of Cells in the Non-dense Core | | | | | *P*^*^ |
|  | cells in  Dense Core | <1% | 1–25% | 26–50% | ≥51% | ≥76% |  |
| Stroma | <1% | 0 | 0 | 0 | 0 |  | <0.001 |
|  | 1–25% | ***2*** | 1 | 0 | 0 |  |  |
|  | 26–50% | ***3*** | ***16*** | 0 | 1 |  |  |
|  | ≥51% | ***0*** | ***35*** | ***6*** | 0 |  |  |
| Epithelium | <1% | 0 | 2 | 0 | 0 |  | <0.001 |
|  | 1–25% | ***29*** | 30 | 0 | 0 |  |  |
|  | 26–50% | ***1*** | *2* | 0 | 0 |  |  |
|  | ≥51% | ***0*** | *0* | *0* | 0 |  |  |
| Adipocytes | <1% | 0 | 0 | 0 | 0 | ***1*** | <0.001 |
|  | 1–25% | 0 | 0 | ***3*** | ***3*** | ***21*** |  |
|  | 26–50% | 0 | 0 | 1 | ***2*** | ***27*** |  |
|  | ≥51-75% | 0 | 0 | 0 | 0 | ***5*** |  |
|  | ≥76% | 0 | 0 | 0 | 0 | 1 |  |
| NOTE: *Italicized bolded numbers for stroma and epithelium reflect the women who have greater stroma and epithelium on dense core compared to non-dense core. Italicized numbers for adipocytes reflect greater proportion of cells on the non-dense vs. dense cores.*  *Proportions of cells in the cores are reflected as integers*  ^*^P-value from signed rank test examining the proportions of stroma, epithelium and adipocytes in the dense vs. non-dense cores. | | | | | | | |
